# Omeprazole Exposure Before Referral in Dogs with Chronic Inflammatory Enteropathy: A Retrospective Observational Single-Centre Study

**DOI:** 10.3390/ani16213378

**Published:** 2026-10-27

**Authors:** Amy Norman, Michelle Walton-Clark

**Affiliations:** 1 Anderson Moores Veterinary Specialists, Linnaeus Veterinary Limited, Winchester SO21 2LL, UK; 2 Eastcott Veterinary Referrals, Linnaeus Veterinary Limited, Edison Business Park, Swindon SN3 3FR, UK

**Keywords:** proton pump inhibitor, gastroprotectants, acid suppression, gastroduodenal ulceration, gastro-oesophageal reflux, primary care, veterinary medicine, stewardship

## Abstract

Omeprazole, a proton pump inhibitor, is widely used in veterinary medicine, although it may be frequently prescribed for conditions in which evidence of benefit is limited. This retrospective observational single-centre study evaluated omeprazole exposure before referral in dogs subsequently diagnosed with chronic inflammatory enteropathy. Medical records from 151 dogs were reviewed, including omeprazole exposure during the six months preceding referral, documented indication, dose and administration frequency. Indications were classified according to American College of Veterinary Internal Medicine consensus recommendations. Overall, 83/151 dogs (55.0%; 95% CI, 47.0–62.7%) had documented omeprazole exposure within six months of referral. Of these, 30/83 (36.1%; 95% CI, 26.6–46.9%) had a documented consensus-consistent indication, whereas 53/83 (63.9%; 95% CI, 53.1–73.4%) did not. Non-specific gastrointestinal signs were the most common documented indication (42/83; 50.6%), followed by suspected gastrointestinal bleeding or gastrointestinal ulceration or erosion (21/83; 25.3%) and suspected gastro-oesophageal reflux or reflux-associated oesophagitis (9/83; 10.8%). Among dogs with a clearly documented once or twice daily oral dosing regimen, once daily administration was more common than twice daily administration (38/65; 58.5% versus 27/65; 41.5%). Omeprazole exposure was common before referral, and a substantial proportion of exposed dogs had no documented indication consistent with ACVIM consensus recommendations. Interpretation was limited by the retrospective design and dependence on the completeness of referring veterinary clinical records.

## Simple Summary

Omeprazole is a medicine that reduces stomach acid and is commonly prescribed to dogs with gastrointestinal signs. However, it is most useful for specific acid-related conditions, such as stomach or intestinal ulceration, upper gastrointestinal bleeding, or inflammation of the oesophagus caused by acid reflux. This study investigated how often dogs were exposed to omeprazole before referral for chronic gastrointestinal disease and whether the reasons for treatment were consistent with current veterinary recommendations. Medical records from 151 dogs were reviewed. More than half of the dogs (83/151; 55.0%) had documented omeprazole exposure during the six months before referral, but only 30/83 (36.1%) had a documented reason for treatment that was consistent with current recommendations. The most common reason for treatment was non-specific gastrointestinal signs, such as vomiting, diarrhoea or reduced appetite, without evidence of ulceration or gastrointestinal bleeding. These findings demonstrate that omeprazole exposure was common before referral in dogs subsequently diagnosed with CIE and that a substantial proportion of exposed dogs had no documented indication consistent with current recommendations.

## 1. Introduction

Omeprazole is a proton pump inhibitor (PPI) and is a potent suppressor of gastric acid secretion widely used in veterinary medicine. In dogs, the PPIs are indicated for the management of acid-related disorders, including gastroduodenal ulceration and erosion (GUE) and gastro-oesophageal reflux-associated oesophagitis [1,2,3]. Omeprazole is a commonly used PPI and has been studied extensively in dogs. When clinically meaningful acid suppression is required, twice-daily administration has been found to be more effective than once-daily treatment [1,4,5].

Despite their established role in the management of acid-related disease, PPIs are frequently prescribed to dogs for conditions in which evidence of clinical benefit is limited or absent [1,6,7,8,9]. In 2018, an American College of Veterinary Internal Medicine (ACVIM) consensus statement on the rational administration of gastrointestinal protectants challenged the routine use of acid-suppressant therapy for several commonly encountered disorders, including non-erosive gastritis, pancreatitis, hepatic disease, and renal disease in the absence of additional risk factors for ulceration or evidence of gastrointestinal haemorrhage [1]. The consensus statement also emphasised that acid suppressants should not be administered solely for the management of vomiting or nausea [1].

Subsequent studies suggest that potentially non-evidence-based PPI prescribing remains common in veterinary medicine. One prospective study evaluated omeprazole use in veterinary specialist care and identified frequent administration in patients without a clearly supported indication [6]. Subsequently, omeprazole prescribing at a UK veterinary teaching hospital was investigated and it was demonstrated that a proportion of prescriptions were inconsistent with evidence-based recommendations, although prescribing practices were found to improve following a clinical audit [7]. In a retrospective study of dogs attending a veterinary teaching hospital in Spain, the proportion of prescriptions classified as appropriate according to the ACVIM consensus recommendations increased from 37% to 74% between 2017 and 2021; nevertheless, unsupported indications remained present [8]. Importantly, the authors cautioned that prescribing patterns identified within a teaching hospital should not be extrapolated to first-opinion veterinary practice. More recently, a survey of 265 veterinarians in Spain found that whilst PPIs were the most commonly used gastroprotectants, only 35.9% of the participants indicated that they made their selection on the basis of scientific evidence [9]. The study documented continued unsupported use of gastroprotectants, with 49.8% of respondents indicating they use PPIs for the management of non-erosive gastritis (*n* = 132), and 44.2% for prevention of GUE in association with NSAID administration [9].

CIE describes a group of gastrointestinal disorders characterised by persistent or recurrent gastrointestinal signs and variable mucosal inflammation [10,11,12]. Vomiting, alterations in appetite, weight loss, and other non-specific gastrointestinal signs frequently occur in affected dogs and could prompt empirical administration of acid-suppressant therapy, despite PPI therapy not being routinely indicated for CIE in the absence of concurrent GUE or oesophagitis [1,10]. Consequently, dogs with CIE may represent a population at particular risk of empirical PPI exposure. To the authors’ knowledge, the frequency, indications, and patterns of omeprazole exposure in dogs with CIE prior to referral have not previously been reported.

Omeprazole administration may also be clinically relevant within CIE patients as acid suppression can alter the canine gastrointestinal microbiota. Dogs with CIE commonly exhibit intestinal dysbiosis, characterised by alterations in microbial composition and diversity and associated changes in microbial metabolites, including reduced faecal short-chain fatty acid concentrations and altered bile acid metabolism [10,13,14,15]. Administration of omeprazole to healthy dogs has been associated with quantitative alterations in the gastrointestinal microbiota, including increased duodenal bacterial abundance and changes in several faecal bacterial taxa [16]. Furthermore, administration of omeprazole in combination with carprofen has been associated with an increased faecal Dysbiosis Index (DI) and faecal calprotectin concentrations in healthy dogs [17]. In contrast, administration of esomeprazole alone produced a numerical but not statistically significant increase in the DI in a more recent study [18], indicating that the effects of PPI administration on the canine intestinal microbiome remain incompletely characterised. The clinical relevance of PPI-associated microbiome alterations in dogs with CIE, in which dysbiosis may already be present, remains unknown; nevertheless, these findings highlight the importance of understanding patterns of acid-suppressant use in this population.

The primary objective of this study was therefore to determine the proportion of dogs subsequently diagnosed with CIE that had been exposed to omeprazole during the six months preceding referral. Secondary objectives were to describe the indications, dose, and dosing frequency of omeprazole treatment, and to determine whether a documented indication consistent with published ACVIM recommendations was present. We hypothesised that omeprazole exposure would be common prior to referral and that a substantial proportion of omeprazole-exposed dogs would have no documented ACVIM-consistent indication for acid suppression.

## 2. Materials and Methods

This was a retrospective, observational, single-centre study enrolling dogs investigated for suspected CIE at a multidisciplinary UK referral hospital. The study population was derived from a previously investigated cohort used to evaluate metronidazole administration before referral in dogs with CIE [19]. The present analysis addressed a distinct research question, focusing on the frequency, indications and prescribing patterns of omeprazole exposure during the six months preceding referral.

The original study population was identified through a search of the electronic medical records for dogs undergoing abdominal ultrasonography between January 2020 and December 2024. Ultrasonographic examinations were undertaken by an ECVIM-boarded diagnostic imaging specialist or a CertAVP in diagnostic imaging supervised by a diplomate, as previously described [19]. The study is reported in accordance with the STROBE-Vet guidelines for reporting observational studies in veterinary medicine [20].

Dogs were eligible for inclusion when they had gastrointestinal clinical signs persisting for greater than three weeks’ duration with no alternative gastrointestinal or extra-intestinal disease causes identified by the attending internal medicine clinician. Given the retrospective nature of the study, investigations were not standardised and were performed at the discretion of the attending internal medicine clinician [19]. Histopathological confirmation was not required for inclusion. Dogs were not subclassified according to subsequent therapeutic response, as the present study specifically evaluated omeprazole exposure prior to referral rather than subsequent treatment response or clinical outcome. Dogs with hypoalbuminaemia (serum albumin < 22 g/L) and evidence of gastrointestinal protein loss were classified as having protein losing enteropathy (PLE) [19].

Dogs were excluded when another disease process was identified as the cause of their gastrointestinal signs, including infectious, neoplastic, endocrine or foreign bodies, or when the pre-referral records were insufficient to establish eligibility. These criteria were consistent with those applied in the previously published cohort [19].

Incomplete medical history was defined as insufficient documentation regarding clinical signs, previous diagnostic investigations, or omeprazole administration during the six months prior to referral. Omeprazole exposure was defined as documented omeprazole prescription or reported administration within the six months prior to referral, irrespective of indication. This information was obtained from referring veterinary records.

The following information was retrieved from the medical records: signalment, weight at referral, date and reason for first presentation to the referral centre, nature of gastrointestinal clinical signs, and final clinical classification as CIE or PLE. Omeprazole exposure within the six months prior to referral was recorded from referring veterinary clinical records, together with the documented indication for treatment. Where available, omeprazole dose, dosing frequency and route of administration were also recorded; missing data for these variables were not considered a reason for exclusion.

Documented indications for omeprazole were subsequently classified according to whether they were consistent with the ACVIM consensus recommendations for rational use of gastrointestinal protectants in dogs and cats [1]. Omeprazole use was considered ACVIM-consistent when the clinical notes documented an indication or clinical signs supporting therapeutic gastric acid suppression. These included suspected or confirmed GUE or upper gastrointestinal haemorrhage (haematemesis, melaena, or anaemia considered clinically compatible with gastrointestinal blood loss), and suspected gastro-oesophageal reflux-associated oesophagitis. Regurgitation alone was not considered sufficient unless the clinical record specifically documented suspected reflux or oesophagitis. Suspected and confirmed gastroduodenal ulceration or upper gastrointestinal haemorrhage were recorded as a combined category during data collection and could not be distinguished retrospectively.

Omeprazole exposure was classified as having no documented ACVIM-consistent indication when it was administered for non-specific gastrointestinal signs without evidence of upper gastrointestinal bleeding or ulceration, including vomiting, diarrhoea, reduced appetite, nausea or abdominal discomfort alone. Other indications classified as non-consistent included suspected or confirmed pancreatitis in the absence of concurrent evidence of GUE, haemorrhagic diarrhoea where there was no evidence to suggest an upper gastrointestinal source, routine prophylactic administration alongside NSAIDs in the absence of documented or suspected GUE, and prophylactic or empirical administration for other conditions for which gastric acid suppression is not supported by current consensus recommendations.

Omeprazole exposure within the six months prior to referral was reviewed alongside the clinical records from the same period to determine the documented reason for treatment. As the indication for omeprazole was not always restated at each repeat prescription, classification was based on the overall clinical context rather than on each prescription in isolation. Where an ACVIM-consistent indication was clearly documented, subsequent prescriptions considered to relate to the same clinical episode or ongoing indication were also classified as ACVIM-consistent, even if the rationale was not repeated in every clinical entry. Concurrent diseases and medications documented in the available clinical record were considered when determining whether an ACVIM-consistent indication for omeprazole exposure was present. Where the clinical record did not contain sufficient information to identify an ACVIM-consistent indication, omeprazole use was classified as having no documented ACVIM-consistent indication rather than assuming an undocumented rationale. Classification of documented indications was performed retrospectively by a single investigator using the predefined criteria described above. Classification was based on review of the available clinical record and was therefore not blinded to other clinical information. As classification was performed by a single investigator; interobserver agreement was not assessed.

No a priori sample-size calculation was performed. Study size was determined by the number of unique dogs meeting the eligibility criteria within the available study population and study period. Each dog contributed a single observation to the study population.

The initial study population comprised 144 eligible dogs identified during the previously published investigation [19]. To identify additional cases for the present analysis, 255 records from the previously screened population were reassessed using the established eligibility criteria. Of these, 150 records were excluded, including 124 that did not meet the eligibility criteria and 26 with insufficient clinical documentation. Following reconciliation with the original cohort to avoid duplicate inclusion, seven additional eligible dogs were identified, resulting in a final study population of 151 dogs (as shown in Figure 1).

The original cohort was assembled to investigate antimicrobial administration before referral; however, eligibility for the present study was based on clinical criteria for CIE and the availability of pre-referral medical records, rather than previous antimicrobial or omeprazole administration. Omeprazole exposure was evaluated separately using the same six-month retrospective assessment period for all included dogs. To reduce selection and information bias, predefined eligibility criteria and a consistent six-month pre-referral exposure window were applied to all dogs. Omeprazole exposure and indication were classified using predefined criteria based on the published ACVIM consensus recommendations. Because data were obtained retrospectively from clinical records, incomplete documentation and misclassification could not be eliminated.

### Statistical Analysis

Continuous data were assessed for normality using the Shapiro–Wilk test and, as they were not normally distributed, are reported as median and interquartile range (IQR). Comparisons between two groups were performed using the Mann–Whitney U test. Categorical data are reported as numbers and percentages, with 95% confidence intervals calculated using the Wilson method for the principal proportions and were compared using Fisher’s exact test. Where data were incomplete, analyses were performed using the available cases for that variable. Age, bodyweight and oral omeprazole dose were analysed as continuous variables and were not categorised for statistical analysis. Breed distribution was assessed descriptively. All tests were two-sided, with statistical significance defined as *p* < 0.05. No adjustment for multiple comparisons was performed. All inferential comparisons were considered exploratory, as the study was primarily descriptive and was not powered to detect associations across multiple subgroups. Secondary exploratory analyses examined omeprazole exposure according to CIE/PLE classification and year of referral.

## 3. Results

A total of 151 unique dogs were included in the final study population (Figure 1), of which 135/151 (89.4%) were classified as having CIE without PLE and 16/151 (10.6%) as having PLE. The median age at diagnosis was 5.7 years (IQR, 2.5–9.8 years; range, 0.5–14.4 years). Eighty-one dogs (53.6%) were male and 70 (46.4%) were female. One hundred dogs (66.2%) were neutered and 51 (33.8%) were entire. Bodyweight was available for 147/151 (97.4%) dogs, with a median bodyweight of 10.7 kg (IQR, 7.3–18.1 kg; range, 2.0–50.2 kg). The most frequently represented breeds were crossbreeds (24/151; 15.9%), Labrador Retrievers (11/151; 7.3%), Cocker Spaniels (10/151; 6.6%), Border Terriers (8/151; 5.3%) and Jack Russell Terriers (8/151; 5.3%). English Springer Spaniels, French Bulldogs and Cockapoos each accounted for 6/151 (4.0%) dogs, with the remaining population comprising a range of other breeds (shown in Supplementary Table S1).

Documented omeprazole exposure within the six months preceding referral was identified in 83/151 (55.0%; 95% CI, 47.0–62.7%) dogs, while 68/151 (45.0%) dogs had no documented omeprazole exposure during this period. The signalment of dogs with and without omeprazole exposure is summarised in Table 1. No statistically significant differences were detected in age, bodyweight, or sex/neuter status between groups. Documented omeprazole exposure was identified in 77/135 (57.0%) dogs with CIE without PLE and 6/16 (37.5%) dogs with PLE. No statistically significant association was detected between CIE/PLE classification and documented omeprazole exposure (*p* = 0.185).

Of the 83 dogs with documented omeprazole exposure during the six months preceding referral, the documented indication was suspected or documented gastrointestinal ulceration or bleeding in 21/83 (25.3%) dogs and suspected gastro-oesophageal reflux or reflux-associated oesophagitis in 9/83 (10.8%) dogs. Non-specific gastrointestinal signs were the documented indication in 42/83 (50.6%) dogs, suspected pancreatitis in 6/83 (7.2%), and prophylactic or other indications in 5/83 (6.0%). Prophylactic or other indications comprised NSAID prophylaxis (*n* = 2), corticosteroid prophylaxis (*n* = 1), prophylactic use associated with a mast cell tumour (*n* = 1), and coughing (*n* = 1).

Overall, 30/83 (36.1%; 95% CI, 26.6–46.9%) dogs had a documented ACVIM-consistent indication for omeprazole exposure, while 53/83 (63.9%; 95% CI, 53.1–73.4%) had no documented ACVIM-consistent indication.

The signalment and disease classification of dogs with documented omeprazole exposure according to the presence or absence of a documented ACVIM-consistent indication are summarised in Table 2. All 30 dogs with documented omeprazole exposure and a documented ACVIM-consistent indication had CIE without PLE, whereas among the 53 dogs without a documented ACVIM-consistent indication, 47/53 (88.7%) had CIE without PLE and 6/53 (11.3%) had PLE. No statistically significant association was detected between disease classification (CIE without PLE vs. PLE) and the presence of a documented ACVIM-consistent indication (*p* = 0.082).

An oral omeprazole dose could be calculated for 75/83 (90.4%) dogs; the remaining 8/83 (9.6%) dogs received intravenous omeprazole and were excluded from the oral dose analysis. The median oral dose per administration was 1.06 mg/kg (IQR, 0.84–1.23 mg/kg; range, 0.55–3.03 mg/kg). There was no statistically significant difference in oral omeprazole dose between dogs treated for ACVIM-consistent and non-consistent indications (*p* = 0.425). Dosing frequency was clearly documented as either SID or BID in 65 dogs, of which three had no recorded dose. No statistically significant association was detected between dosing frequency and the presence of a documented ACVIM-consistent indication (Fisher’s exact test, *p* = 0.588; Table 3).

No statistically significant association was detected between year of presentation and documented omeprazole exposure (*p* = 0.493). Omeprazole exposure was documented in 3/5 (60.0%) dogs presenting in 2020, 25/42 (59.5%) in 2021, 20/35 (57.1%) in 2022, 22/37 (59.5%) in 2023 and 13/32 (40.6%) in 2024. Among dogs with documented omeprazole exposure, no statistically significant association was detected between year of presentation and the presence of a documented ACVIM-consistent indication (*p* = 0.558; Table 4).

## 4. Discussion

The results of the present study demonstrate that documented omeprazole exposure was common in dogs referred for further investigation of CIE, with exposure documented in 55% of dogs within the six months preceding referral. These findings are broadly consistent with previous studies demonstrating frequent use of gastric acid-suppressant therapy in veterinary practice. In a UK referral hospital, gastroprotectants were administered to approximately 40% of hospitalised dogs on both medical and surgical wards, with omeprazole as the most commonly prescribed agent [6]. Similarly, omeprazole was prescribed to 10.9% and 9.5% of all canine patients attending a Spanish veterinary teaching hospital in 2017 and 2021, respectively [8]. More recently, a survey of Spanish veterinarians identified PPIs as the most commonly selected class of acid-suppressant therapy, with omeprazole as the most frequently prescribed drug [9]. Although these populations differ from the present cohort and were not restricted to dogs with CIE, they nevertheless demonstrate the frequency with which PPIs, and omeprazole in particular, are prescribed in veterinary practice. The higher percentage of omeprazole exposure in our study cohort may be related to their diagnosis of CIE and a potentially higher prevalence of gastrointestinal signs when compared to the previous study populations.

In our cohort of dogs diagnosed with CIE, 36.1% of omeprazole-exposed dogs had a documented indication consistent with the ACVIM consensus recommendations. Whilst this is similar to the pre-ACVIM consensus baseline of 37.0% found in a Spanish teaching hospital, it is substantially lower than their post consensus appropriate prescription rate of 74% [8]. UK referral teaching hospitals have also higher rates of compliance, with appropriate indications documented in 91.4% of retrospective cases rising to 98.0% prospectively following clinical audit and staff education in one study and supported indications in 57.1% of internal medicine and 68.4% of surgical hospitalizations in another [6,7]. Authors of referral-based studies have however explicitly cautioned that their high compliance rates benefit from direct specialist oversight, active clinical governance, and academic training, and cannot be extrapolated to first-opinion practice [6,7].

In the current study, 53/83 (63.9%) omeprazole-exposed dogs had no documented ACVIM-consistent indication, with non-specific gastrointestinal signs accounting for 42/83 (50.6%) treated dogs. This closely reflects the prescribing habits reported from a survey of 265 Spanish veterinarians, where 49.8% self-reported administering PPIs for non-erosive gastritis [9]. In an earlier 2021 survey of general practitioners, 89.5% reported prescribing gastroprotectants prophylactically for dogs and cats with non-erosive gastritis [21]. Direct comparison with the present findings is difficult because the survey assessed veterinarians’ self-reported prescribing practices, whereas the present study retrospectively assessed whether an ACVIM-consistent indication was documented in the available clinical records.

Pancreatitis is not considered an indication for routine acid suppression in the absence of concurrent GUE or gastrointestinal bleeding, as vomiting, nausea and abdominal discomfort do not necessarily indicate acid-mediated disease [1,22]. Despite this, suspected pancreatitis accounted for 7.2% (6/83) of omeprazole-exposed dogs in the current study. This is a lower value than shown in previous surveys where 61.3% and 31.3% of veterinarians reported the use of gastroprotectants in the management of pancreatitis [9,21]. However, this difference may reflect the study populations, as dogs with subsequently confirmed pancreatitis were excluded from the current study. Similarly, omeprazole was prescribed prophylactically alongside NSAIDs in only two dogs (2.4%) in the current cohort, compared with 44.2% of respondents in the Spanish survey [9,17]. However, direct comparison is not possible because of differences in study design and population. In particular, NSAID use would generally be avoided in dogs with active gastrointestinal disease, and therefore no conclusions regarding prescribing quality can be drawn from this comparison.

One dog received prolonged omeprazole following surgical correction of a portosystemic shunt. Gastrointestinal mucosal lesions have been documented in dogs with intrahepatic portosystemic shunts, making concern for GUE biologically plausible [23,24]. However, there is currently no evidence that prolonged prophylactic PPI administration following shunt attenuation reduces the risk of GUE or improves clinical outcome [1].

Dose and dosing frequency were secondary descriptive outcomes and were incompletely documented in some dogs. The median oral omeprazole dose in the present study was 1.06 mg/kg per administration, broadly consistent with doses recommended for gastric acid suppression in dogs [1]. Among the 65 dogs with a clearly documented SID or BID regimen, 38/65 (58.5%) received once-daily treatment and 27/65 (41.5%) received twice-daily treatment. Current consensus recommendations favour twice-daily PPI administration when treatment of acid-related GUE is required, supported by pharmacodynamic studies demonstrating more consistent and sustained acid suppression with twice-daily dosing [1,4,5,25]. However, the clinical relevance of dosing frequency depends on the underlying indication, and dosing frequency alone cannot be used to determine whether omeprazole exposure was appropriate. No statistically significant association was detected between dosing frequency and the presence of a documented ACVIM-consistent indication (*p* = 0.588). The consensus statement also recommends gradual tapering following prolonged administration exceeding 3–4 weeks [1]. Potential adverse effects associated with prolonged PPI administration have also been described. Although long-term safety data in dogs remain limited, prolonged PPI exposure has been reported in association with gastric mucosal changes [26,27,28].

Tapering could not be reliably assessed from the retrospective documentation. Prolonged omeprazole administration has also been associated with increased serum gastrin concentrations in dogs [29,30]. In people, prolonged PPI treatment can be followed by rebound acid hypersecretion and the development of acid-related symptoms following withdrawal [31,32]. Comparable clinical evidence in dogs is lacking, and concern regarding rebound acid hypersecretion is largely extrapolated from human medicine. Nevertheless, ACVIM consensus recommendations advise gradual tapering following prolonged PPI administration (>3–4 weeks) because of concern for rebound gastric acid hypersecretion [1].

No statistically significant associations were detected between the presence of a documented ACVIM-consistent indication and the evaluated patient characteristics. No statistically significant differences in age, sex or body weight were detected between dogs with a documented ACVIM-consistent indication and those without a documented ACVIM-consistent indication. Similarly, no statistically significant association was detected between disease classification (CIE without PLE vs. PLE) and the presence of a documented ACVIM-consistent indication. Although all six dogs with PLE were in the group without a documented ACVIM-consistent indication, this association did not reach statistical significance (*p* = 0.082) and should therefore be considered exploratory. The small number of PLE cases precludes meaningful inference regarding an association between disease classification and the presence of a documented ACVIM-consistent indication. These findings did not identify a clear association between the presence of a documented ACVIM-consistent indication and a particular demographic or disease phenotype within this cohort. Previous veterinary prescribing studies have similarly identified the clinical indication and clinician prescribing behaviour as important determinants of whether omeprazole use was considered appropriate, rather than demonstrating a consistent demographic profile associated with appropriate use [8,9].

No statistically significant temporal change in omeprazole exposure or the proportion of dogs with a documented ACVIM-consistent indication was detected over the study period, despite the availability of published ACVIM consensus recommendations promoting rational use of gastrointestinal protectants. However, the small and unequal numbers of dogs included across individual years limit interpretation of these findings. Several factors may have contributed to the high proportion of dogs without a documented ACVIM-consistent indication in the present study. Limited awareness of the consensus recommendations may be one factor, only 43.5% (54/124) of Portuguese general practitioners surveyed were familiar with the 2018 ACVIM consensus statement [21]. More broadly, prescribing decisions may not always be based principally on the available evidence; in a survey of Spanish veterinarians, only 35.9% (95/265) reported selecting gastroprotectants on the basis of scientific evidence, while empirical use for unsupported indications remained common and 94.3% (250/265) expressed a need for further training [9]. Although the present study did not investigate clinicians’ knowledge or reasons for prescribing, these findings identify plausible contributors to the prescribing patterns observed. Studies in referral and teaching hospitals have shown improved appropriateness of omeprazole prescribing following active dissemination of recommendations, education and clinical audit [7,8]. Similar education, audit and feedback initiatives may therefore merit evaluation in primary care practice.

Alterations in intestinal microbial composition and function are well documented in dogs with CIE [13,33,34]. In people, PPI use has been associated with an increased risk of small intestinal bacterial overgrowth (SIBO), potentially because reduced gastric acidity impairs suppression of ingested and upper gastrointestinal bacteria [35,36]. Omeprazole has also been shown to alter gastrointestinal microbial communities in healthy dogs, providing direct veterinary evidence that PPI administration can affect microbial composition [16]. However, the present study did not assess the microbiome or clinical outcomes and therefore cannot establish that omeprazole caused additional dysbiosis or harm in this population. Further studies are required to evaluate the effects of PPIs on the microbiome of dogs with CIE and determine whether these changes influence clinical outcomes.

This study has several limitations. Its retrospective design meant that analyses were dependent on the completeness and accuracy of primary-care clinical records, and undocumented indications for omeprazole administration may therefore have been missed. The distinction between ‘no documented ACVIM-consistent indication’ and inappropriate prescribing is important. As a clinically valid rationale may have existed but not been recorded in the available primary-care notes, this information bias would be expected predominantly to underestimate, rather than overestimate, the proportion of omeprazole-exposed dogs with an ACVIM-consistent indication, although the magnitude of this effect cannot be quantified from the available data. In addition, indication classification was performed by a single investigator, and interobserver agreement was not assessed, introducing the possibility of classification bias. Furthermore, diagnostic investigations were not standardised, and the absence of universal histopathological confirmation may have resulted in variation in diagnostic certainty between cases. Treatment duration and timing relative to individual gastrointestinal episodes were also uncertain in some dogs, while data regarding body weight, dose and administration frequency were incomplete in a proportion of cases. Prescription of omeprazole could not be assumed to equate to administration or owner compliance.

The documented indication for omeprazole exposure was classified retrospectively based on the information documented in the medical record. Consequently, omeprazole exposure may have been misclassified where the clinician’s rationale was not explicitly recorded. In addition, relatively small numbers of dogs were represented within some individual indication categories, limiting the power to detect differences between groups.

The study population substantially overlapped with a previously published cohort of 144 dogs investigated for antimicrobial prescribing prior to referral [19]. The present investigation represents a separate analysis of omeprazole exposure and prescribing indications, with seven additional eligible dogs included in the final population. Use of a previously established study population may have introduced selection bias, particularly if the original screening strategy preferentially identified dogs with more extensive diagnostic investigations, persistent clinical signs or greater prior medication exposure. Consequently, the observed frequency of omeprazole exposure may not be representative of the broader population of dogs with CIE managed in primary-care practice.

Finally, this was a single-centre referral population comprising dogs referred for investigation of CIE. The study population may therefore have been biased towards dogs with more severe, persistent or treatment-refractory gastrointestinal disease and may not be representative of omeprazole prescribing patterns in the wider primary-care CIE canine population. The study was also not designed to assess adverse consequences of PPI exposure, including alterations in the gastrointestinal microbiome, SIBO, NSAID-associated enteropathy or changes in the clinical severity or outcome of CIE; discussion of these potential effects should therefore be interpreted as biologically plausible rather than as evidence of harm demonstrated by the present study.

## 5. Conclusions

To the authors’ knowledge, this is the first study to evaluate documented omeprazole exposure before referral in dogs subsequently diagnosed with CIE. Omeprazole exposure was documented in 55.0% (83/151) of dogs during the six months preceding referral. Among omeprazole-exposed dogs, 36.1% (30/83) had a documented indication consistent with ACVIM consensus recommendations, while 63.9% (53/83) had no documented ACVIM-consistent indication. These findings demonstrate that omeprazole exposure was common in this referral population and that a substantial proportion of exposed dogs had no documented indication consistent with current consensus recommendations. Further studies are required to investigate the factors influencing omeprazole use before referral and to evaluate strategies to support evidence-based use of PPIs in veterinary practice.

## Figures and Tables

**Figure 1:**
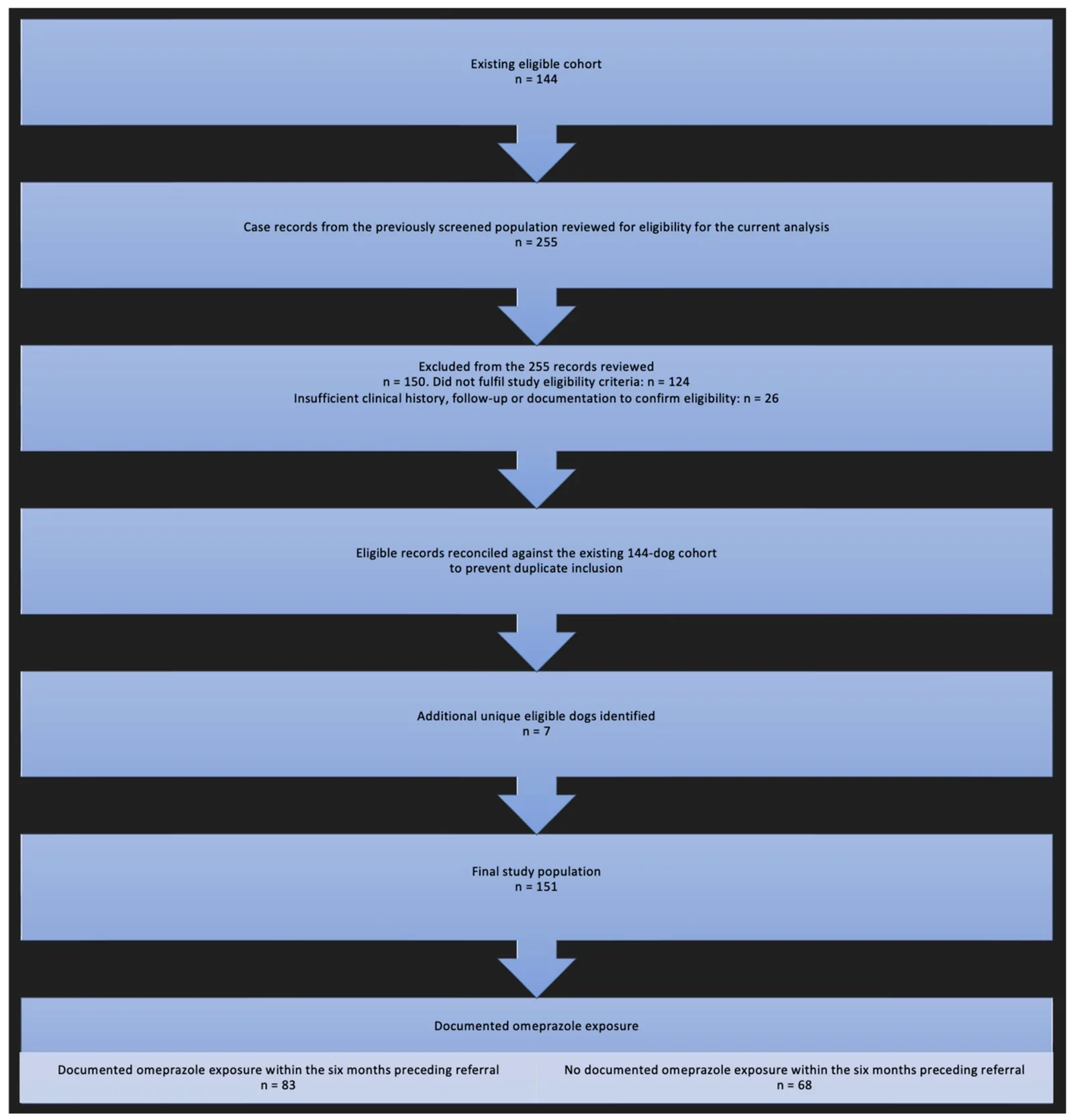
Flow diagram showing construction of the final study population and documented omeprazole exposure within the six months preceding referral.

**Table 1:** Signalment and disease classification of dogs with and without omeprazole exposure within the six months preceding referral.

Variable	Documented Omeprazole Exposure (*n* = 83)	No Documented Omeprazole Exposure (*n* = 68)	*p* Value
Age (years), median (IQR)	5.58 (2.25–9.83)	6.08 (2.69–10.21)	0.526
Weight at referral (kg), median (IQR)	10.10 (6.57–16.95) *^a^	12.50 (8.00–23.05) *^b^	0.137
Sex/neuter status			0.170
Female entire, *n* (%)	5 (6.0%)	5 (7.4%)	
Female neutered, *n* (%)	35 (42.2%)	25 (36.8%)	
Male entire, *n* (%)	17 (20.5%)	24 (35.3%)	
Male neutered, *n* (%)	26 (31.3%)	14 (20.6%)	
Diagnosis			0.185
CIE without PLE, *n* (%)	77 (92.8%)	58 (85.3%)	
PLE, *n* (%)	6 (7.2%)	10 (14.7%)	

*^a^ Bodyweight available for 82/83 dogs, *^b^ Bodyweight available for 65/68 dogs.

**Table 2:** Signalment and disease classification of dogs with documented omeprazole exposure according to the presence or absence of a documented ACVIM-consistent indication.

Variable		ACVIM-Consistent (*n* = 30)	No Documented ACVIM-Consistent Indication (*n* = 53)	*p* Value
Age (years), median (IQR)		4.83 (2.81–9.88)	5.67 (1.96–9.67)	0.936
Weight at referral (kg), median (IQR)		10.00 (5.90–17.80)	10.00 (6.82–16.2)	0.798
Sex/neuter status				0.404
Female entire, *n* (%)		2 (6.7%)	3 (5.7%)	
Female neutered, *n* (%)		15 (50.0%)	20 (37.7%)	
Male entire, *n* (%)		7 (23.3%)	10 (18.9%)	
Male neutered, *n* (%)		6 (20.0%)	20 (37.7%)	
Diagnosis				0.082
CIE without PLE	*n* (%)	30 (100%)	47 (88.7%)	
PLE	*n* (%)	0 (0%)	6 (11.3%)	

**Table 3:** Oral omeprazole dose and dosing regimen according to whether the indication for treatment was consistent with ACVIM consensus recommendations.

Variable	ACVIM-Consistent Indication	No Documented ACVIM Consistent Indication	*p* Value
Oral dose (mg/kg), median (IQR)	1.02 (0.79–1.21), *n* = 25	1.09 (0.85–1.30), *n* = 50	0.425
Dosing frequency (dogs with clearly documented SID or BID regimen), *n* (%)	*n* = 19	*n* = 46	0.588
SID	10 (52.6%)	28 (60.9%)	
BID	9 (47.4%)	18 (39.1%)	

**Table 4:** Omeprazole exposure and consistency of indication with ACVIM consensus recommendations according to year of presentation (percentages are calculated per year).

Year	Total Dogs, *n*	Documented Omeprazole Exposure, *n* (%)	No Documented Omeprazole Exposure, *n* (%)	ACVIM-Consistent Indication, *n* (%)	No Documented ACVIM-Consistent Indication, *n* (%)
2020	5	3 (60.0%)	2 (40.0%)	0 (0%)	3 (100%)
2021	42	25 (59.5%)	17 (40.5%)	10 (40.0%)	15 (60.0%)
2022	35	20 (57.1%)	15 (42.9%)	9 (45.0%)	11 (55.0%)
2023	37	22 (59.5%)	15 (40.5%)	8 (36.4%)	14 (63.6%)
2024	32	13 (40.6%)	19 (59.4%)	3 (23.1%)	10 (76.9%)
Overall	151	83 (55.0%)	68 (45.0%)	30 (36.1%)	53 (63.9%)

## Data Availability

The data presented in this study are available on reasonable request from the corresponding author. The data are not publicly available due to privacy and confidentiality restrictions relating to clinical patient records.

## Supplementary Materials

The following supporting information can be downloaded at https://www.mdpi.com/article/10.3390/ani16213378/s1, Table S1: Breed distributions.

## Abbreviations

The following abbreviations are used in this manuscript:

ACVIM	American College of Veterinary Internal Medicine
BID	Twice daily
CIE	Chronic inflammatory enteropathy
DI	Dysbiosis Index
GUE	Gastroduodenal ulceration and erosion
IQR	Interquartile range
NSAID	Non-steroidal anti-inflammatory drug
PLE	Protein-losing enteropathy
PPI	Proton pump inhibitor
SIBO	Small intestinal bacterial overgrowth
SID	Once daily
