# Supplementary material for: Omeprazole Exposure Before Referral in Dogs with Chronic Inflammatory Enteropathy: A Retrospective Observational Single-Centre Study
Source: Animals (Basel). 2026 Oct 27;16(21):3378. doi: 10.3390/ani16213378 (PMC13762260; doi:10.3390/ani16213378)
Supplement: Supplementary file 1 [file animals-16-03378-s001.zip › animals-4607276-supplementary.pdf]

Table S1. Breed distributions

**Breed**	*n*	**% of study population**
Crossbreed	24	15.9%
Labrador Retriever	11	7.3%
Cocker Spaniel	10	6.6%
Border Terrier	8	5.3%
Jack Russell Terrier	8	5.3%
Cockapoo	6	4.0%
English Springer Spaniel	6	4.0%
French Bulldog	6	4.0%
Dachshund	5	3.3%
Miniature Schnauzer	5	3.3%
Bichon Frise	4	2.6%
Border Collie	4	2.6%
Golden Retriever	4	2.6%
Miniature Dachshund	4	2.6%
West Highland White Terrier	3	2.0%
Boston Terrier	2	1.3%
Cavalier King Charles Spaniel	2	1.3%
Cavapoo	2	1.3%
Lurcher	2	1.3%
Miniature Poodle	2	1.3%
Pomeranian	2	1.3%
Shih Tzu	2	1.3%
Sprocker	2	1.3%
Afghan Hound	1	0.7%
Bassett Hound	1	0.7%

Bearded Collie	1	0.7%
Bulldog	1	0.7%
Cavachon	1	0.7%
Collie X	1	0.7%
English Toy Terrier	1	0.7%
Flat Coated Retriever	1	0.7%
German Shepherd	1	0.7%
Golden doodle	1	0.7%
Greyhound	1	0.7%
Havanese	1	0.7%
Labradoodle	1	0.7%
Maltese	1	0.7%
Norfolk Terrier	1	0.7%
Nova Scotia Duck Tolling	1	0.7%
Parsons Jack Russell Terrier	1	0.7%
Poodle	1	0.7%
Pug	1	0.7%
Schnauzer	1	0.7%
Sheltie	1	0.7%
Springador	1	0.7%
Terrier	1	0.7%
Tibetan Terrier	1	0.7%
Vizsla	1	0.7%
Whippet	1	0.7%
Wire Fox Terrier	1	0.7%
**Total**	**151**	**100.0%**
